# Factors, components and dynamics: investigation of journal self-citation and citation by equal opportunity model

**DOI:** 10.1016/j.heliyon.2022.e10292

**Published:** 2022-08-19

**Authors:** Yangping Zhou

**Affiliations:** Institute of Nuclear and New Energy Technology, Tsinghua University, Haidian, Beijing, 100084, China

**Keywords:** Number of articles, Journal impact factor, Journal self-citation, Equal opportunity model, Multiple citations, Increment effects

## Abstract

The effectivity of journal impact factor (JIF) is questioned in evaluating academic players. Coercive self-citation was widely criticized and Clarivate annually suppressed journals with high journal self-citation (JSC) rates. Recently, some journals significantly increased their articles but their JIFs and JSC rates were diversified which lacks reasonable explanations. Here, we revealed the complexities of the dynamical interactions among different influence factors and different components of journal citation. Journal citation frequencies have strong correlations with JSC rates regarding citable items, significant correlations with numbers of journals' articles and negative significant correlations with JSC rates regarding total citations. Journal citation consists of JSC, intradisciplinary non-JSC and interdisciplinary non-JSC. JSC has the quickest dynamic and interdisciplinary non-JSC has the slowest dynamic while most journals are more cited by interdisciplinary citations. Journal citation is initially decided by the number of citable items. Journal's share (citing articles) in related disciplines influence JSC and intradisciplinary non-JSC positively and negatively, respectively. Multiple citations promoted by the increase in articles, the skewness of the topic profile, preference-related self-citation and anomalous self-citation are of benefit to citation. The complex dynamical interactions result in increment effects of the number of articles acting on JIF, which is stronger for JSC and citable items. The increase in articles also promotes intradisciplinary non-JSC when the journal's shares in related disciplines are low. This process will be reversed after the journal's shares become high enough which may finally decrease its impact factor. It is quicker for the journal with a stronger intradisciplinary citation. The calculational results of the average JIFs of selected journals agree with the statistical results. In addition, we can explain the related situations of some journals. Finally, we suggested that the JSC rate in terms of citable items should be considered for judging the level of JSC.

## 1 Introduction

Clarivate, formerly as Institute for Scientific Information, has released Journal Citation Reports (JCR) for nearly 50 years (Garfield, 1975; Wouters, 2017). The Journal Impact Factor (JIF) in the JCR year, defined as the average value of citations (times cited from the Web of Science Core Collection) to the citable items of a journal published in the previous two years, is used routinely as a key criterion for the evaluation of author, journal, organization, etc.

Recently, JIF is questioned to be an effective indicator of the quality of an article or journal (Wouters et al., 2019; Guo et al., 2021). Firstly, the citation can be manipulated by coercive self-citation (Opthof, 2013; Chorus, 2015; Wilhite et al., 2019) and citation stacking (Heneberg, 2016). Secondly, the citation of an article depends on its discipline context. Thirdly, JIF, the average citation per article published by a journal in a two-year window, cannot exactly indicate the performance of the articles of the journal. The citation distributions in different journals, even in the biggest journals, overlap widely (Larivière et al., 2016).

The evaluation of the players in the scientific area has to balance the various needs of discipline specialty, accuracy, timeliness, maneuverability and transparency since massive authors and articles are now involved in the present journal-discipline context. In 2020, more than 20 million authors published around 10 million publications in more than 53 thousand sources and 155 research areas on Web of Science (WoS). JIF is still the solely available tool that can easily rank players in an ingrained way (Tregoning, 2018; Else, 2019). Clarivate now also adopts the discipline factor as a basic criterion both for ranking journals (JIF Quartile) and determining citation distortions of journals. On the other hand, the motivation, process and result of coercive self-citation were revealed and discussed in detail (Wilhite and Fone, 2012; Mahian and Wongwises, 2015; Chorus and Waltman, 2016; Humphrey et al., 2019; Wilhite et al., 2019). Clarivate annually suppressed journals with high JSC rates and high distortions in the category rank.

In general, journal self-citation (JSC) includes regular self-citation, self-citation based on author self-censoring and coercive self-citation (Chorus, 2015). The last two self-citations can be named anomalous self-citation. Almost every journal introduces its preferred sub-disciplines and topics on the front pages of its homepage. In addition, authors often intend to submit their manuscripts to some preferred journals. The journal-discipline citation context where articles are cited and cite other articles (Zhou, 2021) will be narrowed by these preferences. The self-citation of a journal will increase due to these preferences and we call this part of self-citation preference-related self-citation. Preference-related self-citation is a part of regular self-citation.

Although anomalous self-citation came in for a lot of criticism (Mussard and James, 2017), the effect of self-citation on citation is still an open question. It can be easily inferred that self-citation can increase the citations of articles by promoting their visibility (Francisco et al., 2019). With the same level of JSC, the journals which got higher JIFs will have lower JSC rates in terms of total citations (Gorski et al., 2021). Recent emerging studies indicated the relations between citation and self-citation were usually diversified dependent on what disciplines and/or journals were concerned (Krauss, 2007; Gazni and Didegah, 2021; Jain et al., 2021; Jamalnia and Shokrpour, 2021; Kulczycki et al., 2021; Urlings et al., 2021; Sanfilippo et al., 2021). In addition, longitudinal studies in medical literature showed a decline in the JSC rate in terms of total citations (Delli and Livas, 2018; Delli et al., 2020), the accustomed indicator of JSC at present. In contrast, the JSC rate in terms of citable items increased significantly and had a significant relationship with the total citations (Chorus and Waltman, 2016).

On the other hand, we have to admit that the success of JCR mainly relies on its quantitative description which is a basic character of informatics. Some quantitative approaches were also initialized by the discussion of the relations between self-citation, citation and related influence factors (Yu and Wang, 2007; Galiani and Gálvez, 2019; Brzica, 2021; Huang et al., 2021; Zhou, 2021). In addition, studies were recently promoted for quantitatively predicting the citation of article (Abrishami and Aliakbary, 2019; Huang et al., 2022; Zhao and Feng, 2022; Zhou et al., 2022), author (Ayaz et al., 2018), journal (Rocha-e-Silva, 2016), etc. These quantitative approaches may be essential to establish a robust quantitative model for the citation behaviors of articles and to provide clues and suggestions for the improvement of the present JIF-based evaluation system.

The number of articles increased constantly in the most important academic indexes of WoS, Science Citation Index Expanded (SCIE), Social Sciences Citation Index (SSCI) and Arts and Humanities Citation Index (AHCI) (Zhou, 2021). Some journals increased their numbers of articles significantly but their JIFs and JSC rates were diversified, which lacks reasonable explanations. The complex relations between the number of articles, self-citation and citation were revealed by some studies (Pandita and Singh, 2017; Lariviere et al., 2018; Thelwall and Levitt, 2018; Wilhite et al., 2019.). These relations may be not only size-dependent (van Raan, 2008; Costas et al., 2009) but also size-independent (Javier and Rodrigo, 2018). In addition, cited articles (citable items) and citing articles may act differently on citation (Tahamtan and Bornmann, 2018).

Repeated citations or multiple citations were also discussed by some studies (Copiello, 2019; Giri, 2019). JIF is usually calculated based on times cited of citable items while JSC is available on WoS in terms of citing articles. Repeated citations and multiple citations increased obviously when only regarding self-citation, indicating that repeated citations and multiple citations may significantly affect self-citation and then citation (Lievers and Pilkey, 2012; Copiello, 2019). The ratio of times cited to citing articles (RTCA), an indicator of multiple citations, is significantly correlated with the citations of articles regarding the publishers in Essential Science Indicators (ESI) (Zhou, 2021).

Citations of articles include not only intradisciplinary citation but also interdisciplinary citation. Some studies had been carried out for interdisciplinary citation. The disciplines of educational technology and library and information science had obvious citation linkages (Lund, 2020). The interdisciplinary citation among disciplines related to development and related social sciences was low (Mitra et al., 2020) while the interdisciplinary citation among disciplines related to health and/or place was high (Moon and Pearce, 2020). In addition, the level of interdisciplinary citation positively correlated with JIF (Chen et al., 2021; Petterson et al., 2021).

All mentioned above call for a large-scale and comprehensive study of JSC and journal citation regarding different components of citation and various influence factors. Firstly, the relations between JSC and journal citation and various factors were investigated regarding articles published by all publishers, top publishers and one-journal publishers in ESI. Secondly, the equal opportunity model (EOM) (Zhou, 2021) is applied to calculate the different components of journal citation. Finally, the citation behaviors of journal were discussed regarding the dynamical interactions among components of journal citation and related influence factors. We also suggested an indicator for revealing the level of JSC.

## 2 Materials and methods

### 2.1 Data

The articles (citable items) in SCIE, SSCI and AHCI were initially chosen for this study. Data mining on WoS to gain the bibliometrics information of related journals in ESI was conducted five times (T1–T5, Table 1). The one-journal publisher is the journal publisher that only owns one journal in ESI. The related information of the top publishers can be referred to in the previous study (Zhou, 2021).

**Table 1:** Different data times for data mining.

Data time	Date period	Publisher	Number of publishers/journals	Number of citable items (published year)	Months since end of published year
T1	Mar–Jun 2019	All	1922/13455	2196307 (2016)	30
T2	Jun–Jul 2020	All	1906/13724	2236409 (2017)	31
T3	Jan–Feb 2021	All	1931/13954	2376962 (2018)	26
T4	Jun–Jul 2021	One-journal	50/50	49005 (2018)	31
T5	Oct–Dec 2021	Top 10+one-journal	5/50 + 50/50	34995 + 44297 (2015)	72
		Top 10+one-journal	5/50 + 50/50	39782 + 45626 (2016)	60
		Top 10+one-journal	5/50 + 50/50	40500 + 48583 (2017)	48
		Top 10+one-journal	5/50 + 50/50	48195 + 49904 (2018)	36
		Top 10+one-journal	5/50 + 50/50	50573 + 48556 (2019)	24

In T1–T3, we collected the bibliometrics information of all journals in ESI for overall analysis (see Sections 3.1 and 3.2). In T4 and T5, we selected 100 journals from the journals in ESI for elaborate citation analysis (see Sections 3.3, 3.4, 3.5, 3.6, 3.7). These 100 journals were divided into 10 groups each of which had 10 journals (Table 2). Among them, five groups of journals (No. 1–5) were selected from the top 10 publishers in ESI each of which has 10 randomly-selected journals. Another five groups of journals (No. 6–10) were selected from the one-journal publishers in ESI each of which published most articles. Journals in groups 6 and 7 are only allocated to one WoSC (Web of Science category). Because only one journal published by the one-journal publishers is allocated to five WoSCs, we put it into the group of the journals allocated to four WoSCs.

**Table 2:** Journals used for elaborate citation analysis.

No.	Publisher	Journal	WoSC	No.	Publisher	Journal	WoSC
1	Elsevier	10, randomly selected	1–3	6	one-journal	Top 10 with most articles	1
2	Springer Nature	10, randomly selected	1–3	7	one-journal	Top 11–20 with most articles	1
3	Wiley-Blackwell	10, randomly selected	1–2	8	one-journal	Top 10 with most articles	2
4	IEEE	10, randomly selected	1–4	9	one-journal	Top 10 with most articles	3
5	Walter de Gruyter	10, randomly selected	1–4	10	one-journal	Top 10 with most articles	4–5

For the indicators with obvious cumulative effects, e.g. total citations (citing articles or times cited) and total citations per citable items, the values of them were influenced by the time interval between the date when the citable items were published and the data time. When comparing these indicators in different data times, the differences in the time intervals should be considered if these differences were big. Because the accurate calculation of the time intervals is a task with a huge workload, we adopt the time interval in months between the end of the published year of citable items and the end of the data time. E.g. the time intervals of T2 and T3 are 31 months and 26 months respectively. The value of total citations in T3 can be multiplied by (31–26)/26 when comparing it with that in T2.

### 2.2 Configurations and indicators

#### 2.2.1 Citation counter and Web of Science category

Often, two kinds of citation counters are available, citing articles and times cited. Since only citing articles can be achieved on WoS to get their journals, citing articles are used to measure the JSC rate. In this article, citation usually indicates citing articles unless additional hints. Here, we adopted WoSC to indicate the discipline issues of journals and articles.

#### 2.2.2 Anomalous self-citation and preference-related self-citation of journal

JSC can be categorized as regular self-citation, self-citation based on author self-censoring and coercive self-citation (Chorus, 2015).

Different from coercive self-citation, self-citation based on author self-censoring is motivated by the author himself which also deviates from the real academic motivation for citing articles. E.g., before submitting a manuscript to a journal, the author may add some unnecessary references to it because they can improve the probability of passing the review of editors and reviewers. The last two kinds of self-citation are anomalous self-citation. Here, we use anomalous self-citation as a factor to influence journal self-citation and citation rather than coercive self-citation.

We also consider a kind of regular self-citation of journal, preference-related self-citation. When visiting the homepage of a journal, we usually can find unambiguous descriptions of its preferred sub-disciplines and topics on its front pages. A journal's preference for some sub-disciplines and topics will narrow the journal-discipline citation context where its articles are cited and cite other articles. In addition, authors usually submit their manuscripts to one or several journals though many journals may be available in the related disciplines. Based on the EOM model (Zhou, 2021), the JSC rate will be initially determined by the ratio of the journal's articles to the total articles in the related disciplines. JSC will be increased by the journal's preference for related sub-disciplines and topics and the author's preference for related journals. We call this part of JSC preference-related self-citation.

#### 2.2.3 Journal citation frequency

Journal citation frequency (JCF) indicates the citable item in a year and their received citations in the concerned time window. Here, three kinds of JCFs are calculated according to citing articles (Eq. (1)), times cited (Eq. (2)) and non-JSCs (Eq. (3)) of the citable items of a journal published in a year:

(1) $$JCF=\frac{C}{n_{A}}\;$$

(2) $$JCF_{T}=\frac{T}{n_{A}}$$

(3) $$JCF_{NS}=\frac{C-JSC}{n_{A}}$$

where, C is citing articles of the citable items of a journal published in a year; $n_{A}$ is the number of the citable items of a journal published in a year; T is times cited of the citable items of a journal published in a year.

#### 2.2.4 JSC rate

At present, the JSC rate regarding the journal's total citations ($JSCR_{C}$) are usually considered that indicates the ratio of its JSCs to its total citations. Another kind of journal self-citation rate regarding citable items ($JSCR_{A}$) indicates the ratio of its JSCs to its citable items. Various JSC rates are calculated according to Eqs. (4) and (5):

(4) $$JSCR_{C}=\frac{JSC}{C}$$

(5) $$JSCR_{A}=\frac{JSC}{n_{A}}$$

#### 2.2.5 Multiple citations and RTCA

Here, we define multiple citations of a journal as the phenomenon that a citing article cites more than one article published by the journal in the concerned time window. The self-citations are only available in terms of the citing articles on the WoS and the JIF is calculated regarding the number of times cited for citable items in the two-year window. And therefore, the multiple citations of a journal have an effect to conceal the real level of its self-citations. In addition, when an article cited more than one article from a journal in a short time window, this may indicate a high possibility that the cited articles concerned the same topic. The level of the multiple citations of a journal may also reveal the skewness of its topic profile. The RTCA of a journal ($\tau_{J}$) shows its level of the multiple citations that is calculated by Eq. (6) as follows:

(6) $$\tau_{J}=\frac{T}{C}$$

### 2.3 Calculation of journal citations based on EOM

#### 2.3.1 Values of various citations

The citations of a journal include JSCs, intradisciplinary non-JSCs ($C_{RN}$) and interdisciplinary non-JSCs ($C_{EN}$). Various citations of a journal can be calculated by Eqs. (7), (8), and (9) as follows by modifying related equations based on the EOM model in the previous study (Zhou, 2021):

(7) $$JSC=\frac{2f_{JS}b}{\tau_{J}\left(1+\tau_{J}\right)}\overset{m}{\underset{j=1}{\sum }}\left(SP_{j}\underset{i}{\sum }\left(r_{i}p_{JS,i,j}\right)\right)$$

(8) $$C_{RN}=\frac{f_{RN}b}{\tau_{J}}\overset{m}{\underset{j=1}{\sum }}\left(SP_{j}\underset{i}{\sum }\left(r_{i}\left(1-p_{JS,i,j}\right)\right)\right)$$

(9) $$C_{EN}=\frac{2f_{EN}b}{\left(1+\tau_{J}\right)}\overset{m}{\underset{j=1}{\sum }}\left(SP_{j}\right)$$

where, $f_{JS}$ is the coefficient of JSC; b is the average references of articles; j is the serial number of a WoSC; m is the number of WoSCs; $SP_{j}$ is the number of citable items of a journal allocated to the *j*th WoSC; i is the published year of citing articles; $r_{i}$ is the ratio of citations from the *i*th year to total citations; $p_{JS,i,j}$ is the ratio of JSCs to intradisciplinary citations (citing articles published in the *i*th year and allocated to the *j*th WoSC); $f_{RN}$ is the coefficient of intradisciplinary non-JSC; $f_{EN}$ is the coefficient of interdisciplinary non-JSC.

The JSCs of an article are proportional to the ratio of articles in its journal to total articles in its allocated disciplines (WoSCs) in terms of possible citing articles. And therefore, Eq. (10) is applied to obtain the related values in Eqs. (7) and (8):

(10) $$p_{JS,i,j}=\frac{SP_{i,j}}{P_{i,j}}$$

where, $SP_{i,j}$ is the number of articles of a journal published in the *i*th year and allocated to the *j*th WoSC; $P_{i,j}$ is the number of articles published in the *i*th year and allocated to the *j*th WoSC.

In general, the calculation of different components of journal citation considers three aspects. The basic aspect is the parts of b∑(·) in Eqs. (7), (8), and (9) which sum the related citations for the citable items in their allocated disciplines (WoSCs). The other two aspects are two kinds of adjustments. One is for different components of journal citation, $f_{JS}$, $f_{RN}$ and $f_{EN}$. Another is for different citation counters, times cited and citing articles.

The right parts of Eqs. (7), (8), and (9) originally calculate the times cited of a journal and the left parts of them are based on citing articles. Because multiple citations increased obviously when only regarding self-citation (Lievers and Pilkey, 2012; Copiello, 2019), we assumed that the ratio of times cited to citing article is highest for JSCs and lowest for interdisciplinary non-JSCs. And therefore, Eqs. (7), (8), and (9) are here corrected by different coefficients, $2/\left(\tau_{J}\left(1+\tau_{J}\right)\right)$, $1/\tau_{J}$.and $2/\left(1+\tau_{J}\right)$, respectively.

#### 2.3.2 Equations for linear regression

We can obtain Eq. (11) of a journal according to Eqs. (7), (8), and (9):

(11) $$\frac{C}{C_{EN}}-1=\frac{2f_{JS}-f_{RN}\left(1+\tau_{J}\right)}{2f_{EN}}\left(\frac{\sum_{j=1}^{m}\left(SP_{j}\sum_{i}\left(r_{i}p_{JS,i,j}\right)\right)\;}{\tau_{J}\sum_{j=1}^{m}\left(SP_{j}\right)}\right)+\frac{\left(1+\tau_{J}\right)f_{RN}}{2\tau_{J}f_{EN}}$$

The ratios of intradisciplinary citations to interdisciplinary citations of most journals are often higher than or close to 1 while the ratios of some journals are quite smaller than 1. This may indicate the citation behaviors of those two kinds of journals are different. The above-mentioned require an adjustment ($r_{1}$) for the ratio of total citations to interdisciplinary citations of journals. $r_{1}$ can be obtained by Eq. (12):

(12) $$r_{1}=1\;\left(\frac{C_{EN}}{C_{R}}>0.8\right),\;0.6\;\left(0.4<\frac{C_{EN}}{C_{R}}\leq 0.8\right),\;0.4\left(\frac{C_{EN}}{C_{R}}\leq 0.4\right)$$

where, $C_{R}$ is intradisciplinary citations of a journal.

Then, Eq. (11) can be transformed as Eqs. (13), (14), and (15):

(13) $$y=\frac{2f_{JS}-f_{RN}\left(1+\tau_{J}\right)}{2f_{EN}}x+\frac{\left(1+\tau_{J}\right)f_{RN}}{2\tau_{J}f_{EN}}$$

(14) $$y=r_{1}\frac{C}{C_{EN}}-1$$

(15) $$x=\frac{\sum_{j=1}^{m}\left(SP_{j}\sum_{i}\left(r_{i}p_{JS,i,j}\right)\right)\;}{\tau_{J}\sum_{j=1}^{m}\left(SP_{j}\right)}$$

In Eq. (13), there are two unknown numbers, $f_{JS}/f_{EN}$ and $f_{RN}/f_{EN}$, whose values can be obtained by a regression fitting of related values of some selected journals. We selected 10 groups of journals for the regression fitting whose bibliometrics information was obtained in T5 for the citable items (articles) published in 2018 (Table 2).

## 3 Results

### 3.1 Bibliometrics information of journals and publishers

**Figure 1:**
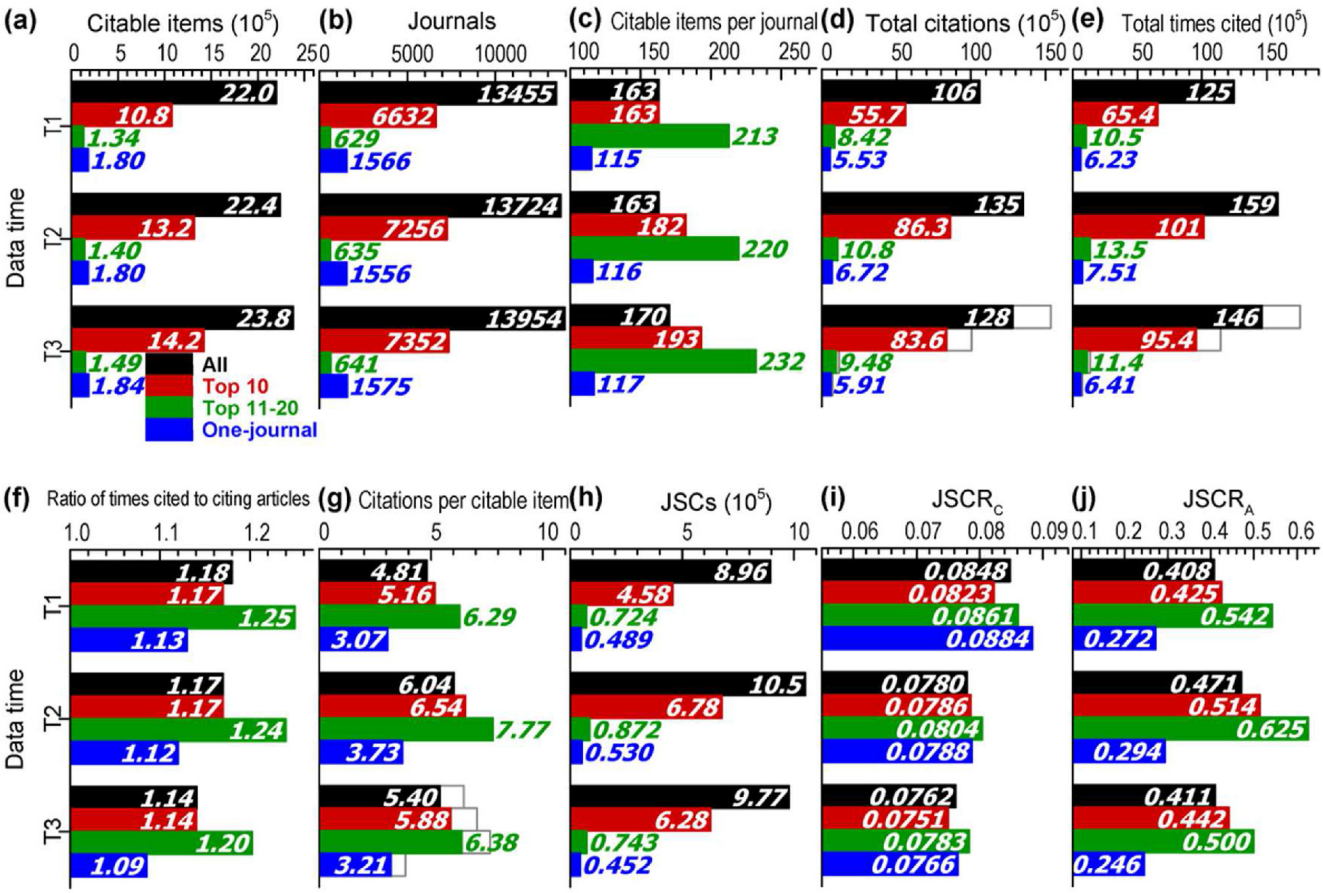
Bibliometrics information of journals regarding different publishers. **(a)** Citable items. **(b)** Journals. **(c)** Citable items per journal. **(d)** Total citations. **(e)** Total times cited. **(f)** Ratio of times cited to citing articles. **(g)** Citations per citable item. **(h)** JSCs. **(i)** JSCR_C_**(j)** JSCR_A_ (T1: data time in 2019. T2: data time in 2020. T3: data time in 2021. JSC: journal self-citation. JSCR_C_: journal self-citation rate (total citations). JSCR_A_: journal self-citation rate (citable items).).

Bibliometrics information of the journals regarding different publishers in ESI is shown in Figure 1. Citable items and journals increased constantly in 2016–2018. Citations and citations per citable item also increased constantly if considering the differences in time intervals in different data times (Table 1 and gray frames in Figures 1(d), (e) and (g)). The ratio of time cited to citing articles was stable in T2 and decreased in T3. In general, JSC rates decreased while the JSC rate in terms of citable items increased in T2.

In Figure 1, only the values of the JSC rates in terms of total citations for different publishers are close. The top 10 publishers published around half of all articles and journals in ESI and got around 60% of citations and JSCs. The top 11–20 publishers had the highest values of other indicators, average citable items (articles) of journals, citations per citable item, the RTCA and the JSC rate in terms of citable items while the one-journal publishers had the lowest values.

### 3.2 Relations between JSC, journal citation and influence factors

Relations between JSC rates, journal citations and influence factors of all journals in ESI are revealed by grouping journals to different publishers (T1–T3, Table 3). Since there was no data in the normal data distribution, the Spearman correlation coefficient was used for correlation analysis.

**Table 3:** Relations between JSC, journal citation and influence factors of different journals in ESI.

Data time	Publishers	*rho*	JCF	JCF_NS_	JSCR_C_	JSCR_A_	$n_{A}$
T1	All/Top10/Top11-20/one-Journal	JSCR_C_	-.22∗∗/-.32∗∗/-.37∗∗/-.22∗∗	-.28∗∗/-.40∗∗/-.46∗∗/-.33∗∗	∖	.51∗∗/.58∗∗/.53∗∗/.58∗∗	.12∗∗/.08∗∗/.03/.07∗∗
	All/Top10/Top11-20/one-Journal	JSCR_A_	.62∗∗/.51∗∗/.54∗∗/.57∗∗	.56∗∗/.44∗∗/.45∗∗/.47∗∗	.51∗∗/.58∗∗/.53∗∗/.58∗∗	∖	.34∗∗/.24∗∗/.19∗∗/.20∗∗
	All/Top10/Top11-20/one-Journal	$n_{A}$	.27∗∗/.21∗∗/.14∗∗/.18∗∗	.26∗∗/.20∗∗/.13∗∗/.16∗∗	.12∗∗/.08∗∗/.03/.07∗∗	.34∗∗/.24∗∗/.19∗∗/.20∗∗	∖
	All/Top10/Top11-20/one-Journal	$\tau_{J}$	.30∗∗/.13∗∗/.30∗∗/.24∗∗	.26∗∗/.08∗∗/.23∗∗/.17∗∗	.42∗∗/.50∗∗/.46∗∗/.43∗∗	.60∗∗/.57∗∗/.69∗∗/.57∗∗	.34∗∗/.29∗∗/.23∗∗/.27∗∗
T2	All/Top10/Top11-20/one-Journal	JSCR_C_	-.22∗∗/-.32∗∗/-.38∗∗/-.16∗∗	-.28∗∗/-.39∗∗/-.46∗∗/-.30∗∗	∖	.51∗∗/.58∗∗/.55∗∗/.59∗∗	.13∗∗/.09∗∗/.07/.08∗∗
	All/Top10/Top11-20/one-Journal	JSCR_A_	.61∗∗/.51∗∗/.50∗∗/.63∗∗	.56∗∗/.44∗∗/.41∗∗/.50∗∗	.51∗∗/.58∗∗/.55∗∗/.59∗∗	∖	.35∗∗/.26∗∗/.22∗∗/.20∗∗
	All/Top10/Top11-20/one-Journal	$n_{A}$	.25∗∗/.21∗∗/.13∗∗/.18∗∗	.23∗∗/.20∗∗/.11∗∗/.16∗∗	.13∗∗/.09∗∗/.07/.08∗∗	.35∗∗/.26∗∗/.22∗∗/.20∗∗	∖
	All/Top10/Top11-20/one-Journal	$\tau_{J}$	.29∗∗/.17∗∗/.23∗∗/.34∗∗	.25∗∗/.12∗∗/.17∗∗/.20∗∗	.44∗∗/.51∗∗/.50∗∗/.42∗∗	.61∗∗/.61∗∗/.69∗∗/.57∗∗	.35∗∗/.34∗∗/.28∗∗/.26∗∗
T3	All/Top10/Top11-20/one-Journal	JSCR_C_	-.25∗∗/-.31∗∗/-.35∗∗/-.21∗∗	-.30∗∗/-.37∗∗/-.42∗∗/-.30∗∗	∖	.50∗∗/.56∗∗/.54∗∗/.59∗∗	.15∗∗/.11∗∗/.16∗∗/.10∗∗
	All/Top10/Top11-20/one-Journal	JSCR_A_	.62∗∗/.54∗∗/.55∗∗/.58∗∗	.57∗∗/.48∗∗/.48∗∗/.50∗∗	.50∗∗/.56∗∗/.54∗∗/.59∗∗	∖	.36∗∗/.26∗∗/.22∗∗/.21∗∗
	All/Top10/Top11-20/one-Journal	$n_{A}$	.25∗∗/.20∗∗/.09∗/.17∗∗	.24∗∗/.19∗∗/.08/.16∗∗	.15∗∗/.11∗∗/.16∗∗/.10∗∗	.36∗∗/.26∗∗/.22∗∗/.21∗∗	∖
	All/Top10/Top11-20/one-Journal	$\tau_{J}$	.14∗∗/-.01/-.03/.12∗∗	.11∗∗/-.04∗∗/-.06/.06∗	.46∗∗/.47∗∗/.40∗∗/.50∗∗	.49∗∗/.38∗∗/.33∗∗/.49∗∗	.46∗∗/.47∗∗/.50∗∗/.35∗∗

Spearman, two-tailed.

∗ significant at level of 0.05.

∗∗ significant at level of 0.01.

All related correlations of journals regarding different publishers and data times are close except for the correlations between different citations and the RTCA ($\tau_{J}$) in T3.

The JSC rate in terms of total citations had negative significant correlations with citation and non-JSC but it had a strong significant correlation with the JSC rate in terms of citable items and a significant correlation with the RTCA. The JSC rate in terms of citable items had strong significant correlations with citation, non-JSC and the RTCA.

The number of articles had significant correlations with citation, non-JSC and the RTCA. The RTCA also had significant correlations with citation and non-JSC except for those of the top 20 publishers in T3. In contrast, correlations between the number of articles and the RTCA became stronger in T3.

### 3.3 Calculational results of ratio of interdisciplinary citations to total citations

**Figure 2:**
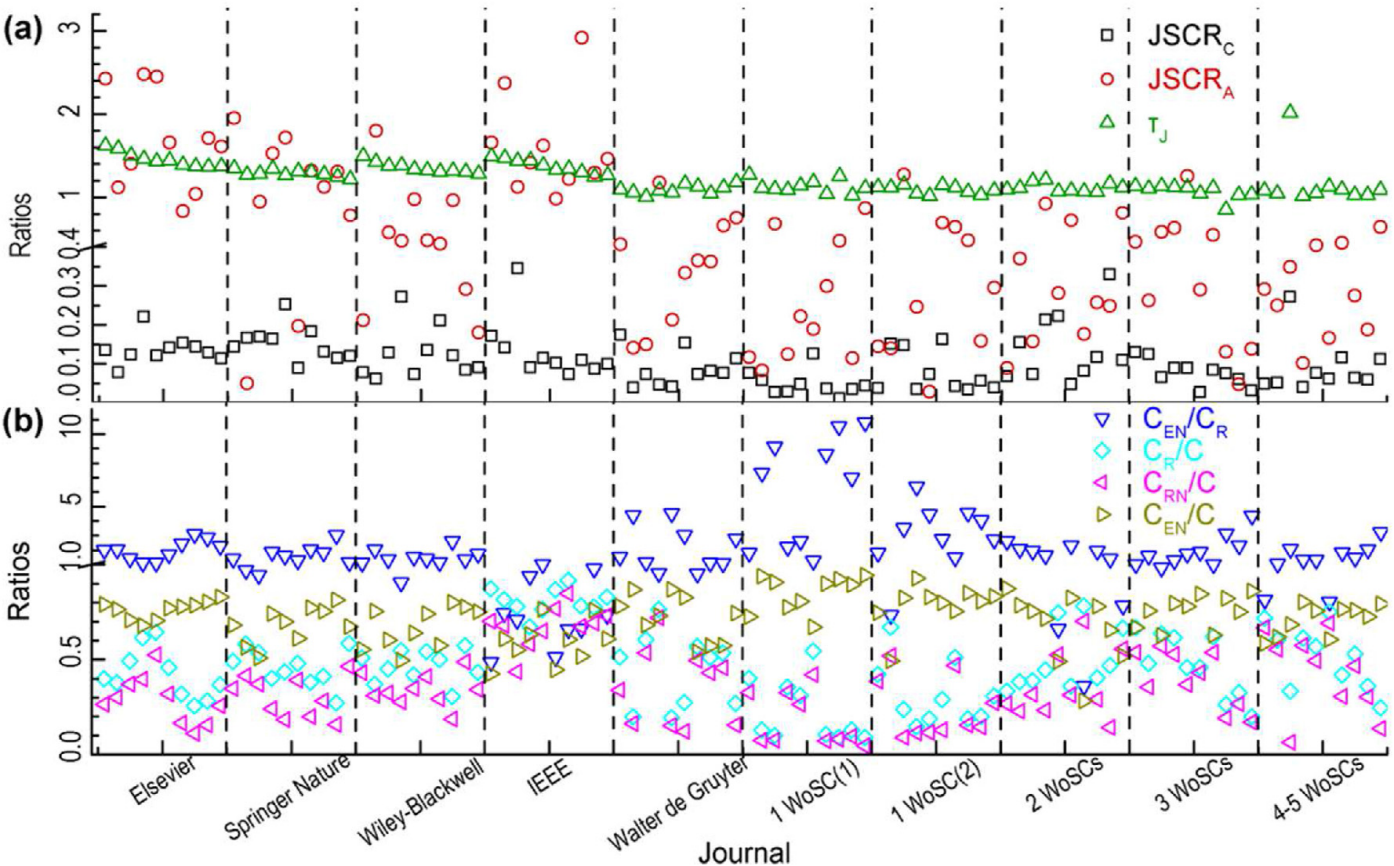
Bibliometrics information of 100 selected journals obtained in T5 for citable items published in 2018. **(a)** JSCR_C_, JSCR_A_ and $\tau_{J}$. **(b)** C_EN_/C_R_, C_R_/C, C_RN_/C and C_EN_/C (T5: data time in October–December 2021. JSCR_C_: journal self-citation rate (total citations). JSCR_A_: journal self-citation rate (citable items). $\tau_{J}$: ratio of times cited to citing articles. C_EN_: interdisciplinary non-self-citations. C_R_: intradisciplinary citations. C_RN_: intradisciplinary non-self-citations. C: total citations. WoSC: Web of Science category).

Bibliometrics information of the 100 selected journals for the regression fitting is shown in Figure 2 which was obtained in T5 for citable items published in 2018 (Table 1). The JSC rates in terms of total citations and the RTCAs are close for different journals. The JSC rate in terms of citable items is quite different for different journals whose values are ranged from around 0–3. The ratios of interdisciplinary citations to intradisciplinary citations are higher than 1 for most journals. Journals published by IEEE had strong intradisciplinary citations while the top 10 journals published by the one-journal publishers got more interdisciplinary citations if they were only allocated to one WoSC. Because many journals are allocated to more than one WoSC, some citations from these journals may simultaneously belong to interdisciplinary citations and intradisciplinary citations of a journal. And therefore, the sum of the ratio of interdisciplinary citations to total citations and the ratio of intradisciplinary non-JSCs to total citations is usually bigger than 1 for a journal.

The results of the regression fitting of Eq. (13) are shown in Table 4 whose data came from the related 10 groups of journals (Table 2 and Figure 2). The related small fitting errors related to $f_{RN}/f_{EN}$ indicate the uniform behaviors of none-JSC of the concerned journals. The big fitting errors related to $\left(f_{JS}-f_{RN}\right)/f_{EN}$ indicate the big difference in the behaviors of self-citation of the concerned journals which may be mainly due to their differences in preference-related self-citation and anomalous self-citation.

**Table 4:** Fitting results based on EOM.

No.	$\frac{\left(1+\tau_{J}\right)f_{RN}}{2\tau_{J}f_{EN}}$		$\frac{2f_{JS}-f_{RN}\left(1+\tau_{J}\right)}{2f_{EN}}$		No.	$\frac{\left(1+\tau_{J}\right)f_{RN}}{2\tau_{J}f_{EN}}\;$		$\frac{2f_{JS}-f_{RN}\left(1+\tau_{J}\right)}{2f_{EN}}$	
	Value	SE	Value	SE		Value	SE	Value	SE
1	0.40	0.08	-1.44	1.37	6	0.16	0.08	0.58	1.16
2	0.62	0.10	-3.26	2.13	7	0.20	0.04	1.03	1.41
3	0.49	0.13	1.57	3.24	8	0.34	0.05	0.06	0.74
4	0.28	0.09	-4.20	4.17	9	0.31	0.07	3.12	2.05
5	0.48	0.11	-0.08	4.91	10	0.39	0.05	5.79	2.29

SE: standard error.

**Figure 3:**
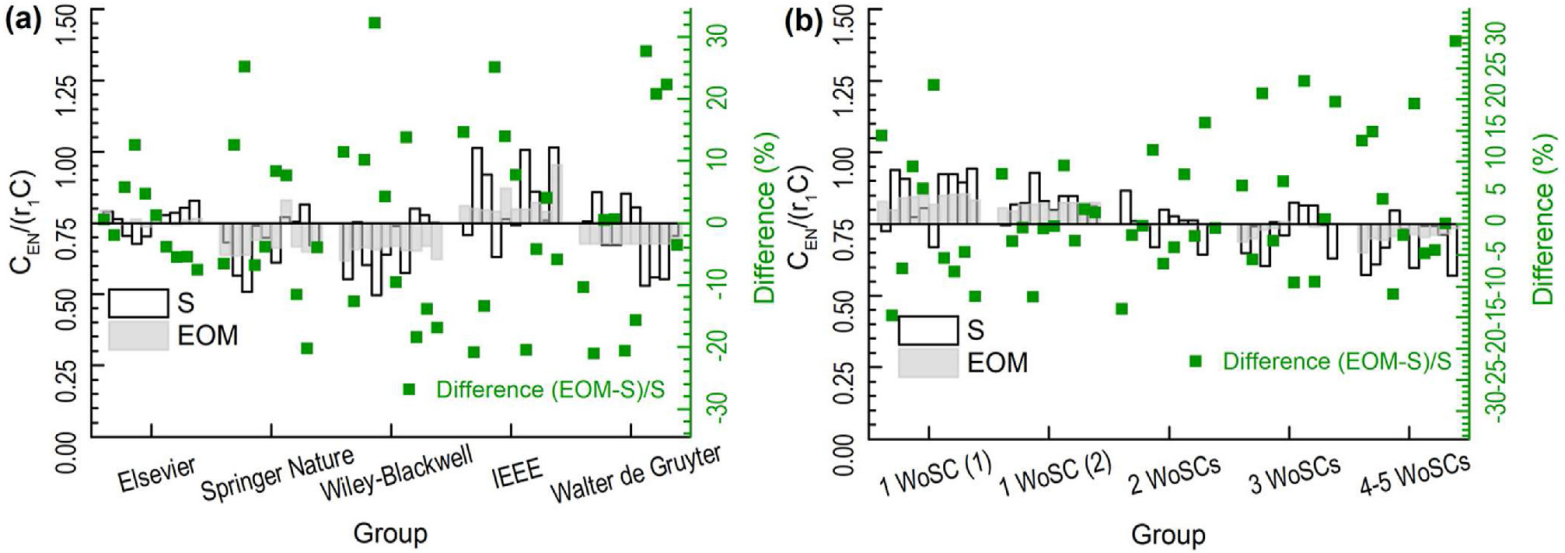
Comparisons of results based on statistics and EOM for ratios of interdisciplinary non-self-citations to adjusted total citations of 10 groups of selected journals. **(a)** Selected journals from top 10 journal publishers in ESI. **(b)** Selected journals from one-journal publishers in ESI (C: citations of a journal. C_EN_: interdisciplinary non-self-citations of a journal. r_1_: adjustment coefficient for ratio of total citations to interdisciplinary citations. S: statistics. EOM: equal opportunity model. WoSC: Web of Science category).

In addition, the ratio of interdisciplinary citations to adjusted total citations, $C_{EN}/\left(r_{1}C\right)$, obtained by using the fitting results, are compared with those obtained by statistics (Figure 3). The differences are within the range of ±35% and the majority of them are within the range of ±20%. It should be noted that the numbers of articles in a journal are usually from around one hundred to several thousand in a year. This makes the influence of citations of the individual article on the citations of a journal more sensitive than those on the citations of a publisher in the previous study (Zhou, 2021). The differences between the selected journals of Elsevier are the smallest which may be explained by its more articles per journal. The more articles a journal has, the smaller the influence of an individual article on the citations of a journal is. The differences between the selected journals of Walter de Gruyter are the biggest. The average articles per journal of Walter de Gruyter were the smallest where the influence of the citation of the individual article on the citation of a journal is the most sensitive. In addition, when the journals were grouped according to the numbers of their WoSCs, the differences became smaller.

### 3.4 Dynamics of self-citations, citations and influence factors of journal

**Figure 4:**
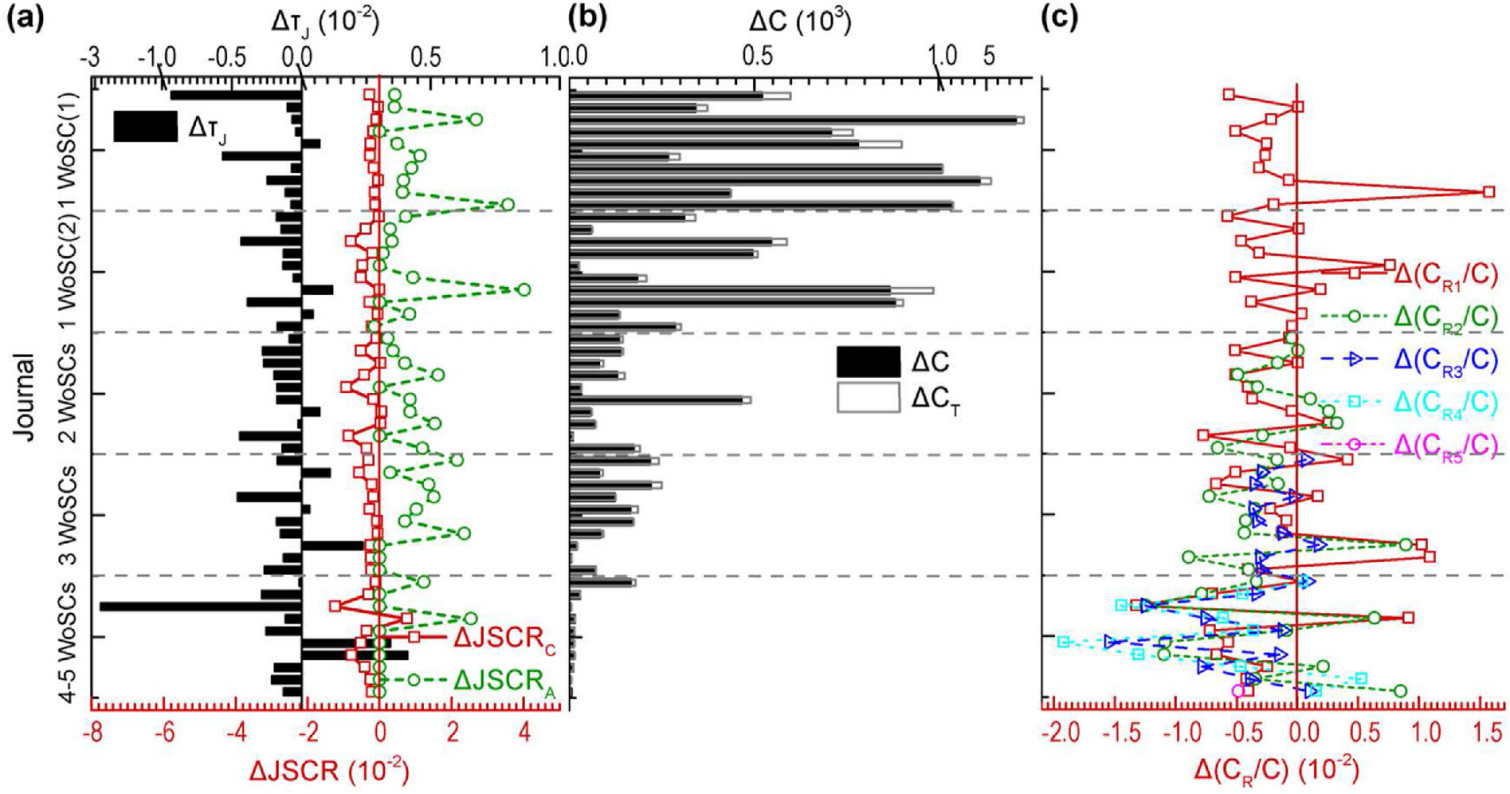
Differences in indicators and citations between T4 and T5 (Δ = T5-T4) regarding citable items published by 50 selected one-journal publishers in 2018. **(a)** Δ $\tau_{J}$ and ΔJSCR. **(b)** ΔC. **(c)** Δ(C_R_/C) (T4: data time in June–July 2021. T5: data time in October–December 2021. $\tau_{J}$: ratio of times cited to citing articles. JSCR: journal self-citation rate. JSCR_C_: journal self-citation rate (total citations). JSCR_A_: journal self-citation rate (citable items). C: citations (citing articles). C_T_: citations (times cited). C_Ri_: intradisciplinary citations of the *i*th WoSC to which a journal is allocated.).

We compared citations and related indicators of the 50 selected journals of the one-journal publishers in T4 and T5 (Figure 4). Here, the citable items were published in 2018. The RTCAs in most journals decreased slightly with time (Figure 4(a)). Most journals have small decreases in the JSC rate in terms of total citations and have big increases in the JSC rate in terms of citable items. Of course, the citations of each journal increased (Figure 4(b)). The ratio of intradisciplinary citations to total citations decreased for most journals (Figure 4(c)).

**Figure 5:**
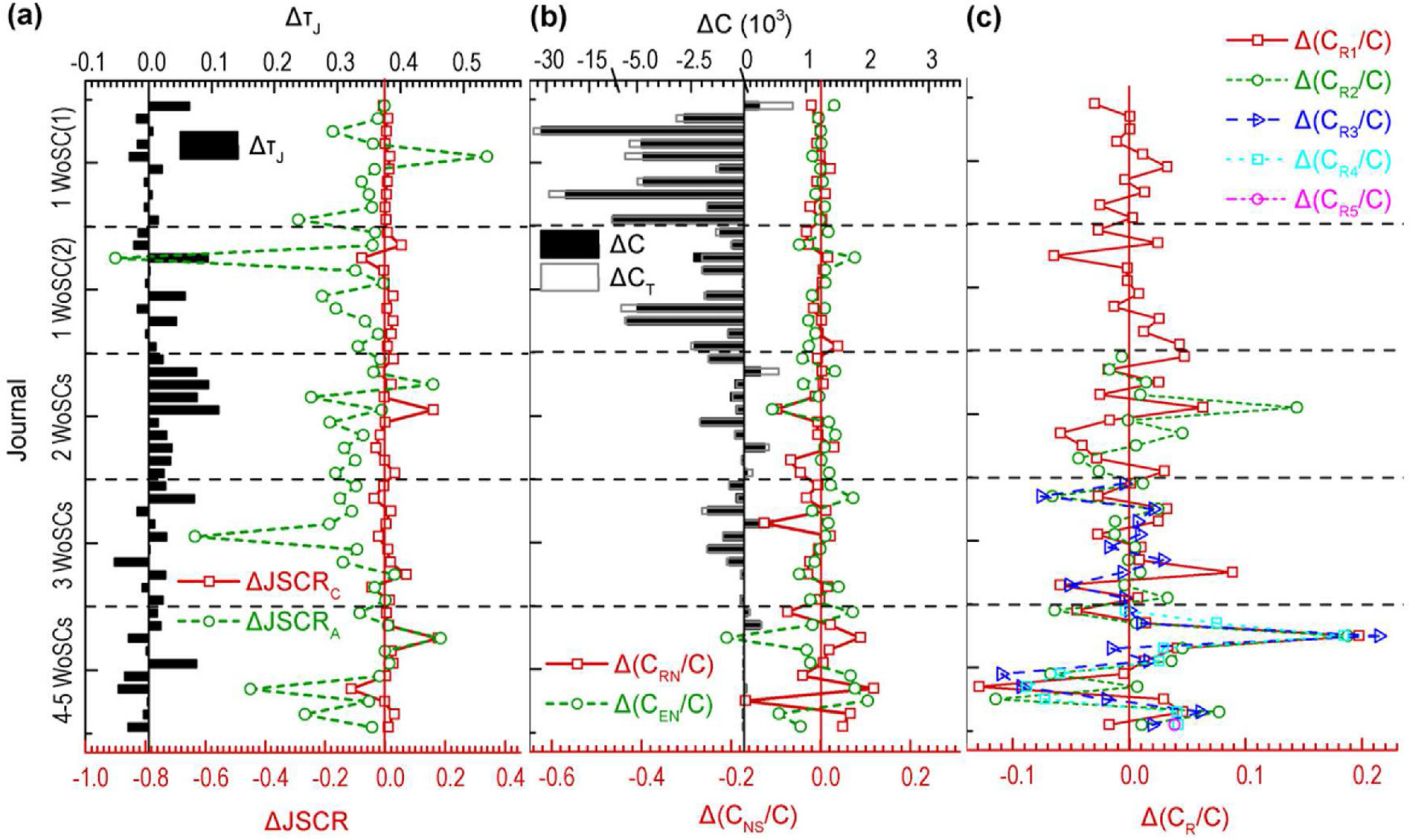
Differences in citations and indicators in T5 between citable items published in 2018 and 2019 (Δ = 2019–2018) by 50 selected one-journal publishers. **(a)** Δ $\tau_{J}$ and ΔJSCR. **(b)** ΔC and Δ(C_NS_/C). **(c)** Δ(C_R_/C) (T5: data time in October–December 2021. $\tau_{J}$: ratio of times cited to citing articles. JSCR: journal self-citation rate. JSCR_C_: journal self-citation rate (total citations). JSCR_A_: journal self-citation rate (citable items). C: citations (citing articles). C_T_: citations (times cited). C_NS_: non-self-citations. C_RN_: intradisciplinary non-self-citations. C_EN_: intradisciplinary non-self-citations. C_Ri_: intradisciplinary citations of *i*th WoSC to which a journal is allocated.).

We also compared citations and related indicators of the 50 selected journals of the one-journal publishers in T5 whose citable items were published in 2018 and 2019 (Figure 5). The differences in RTCAs are not obvious (Figure 5(a)). Citable items of most journals published in 2019 have higher JSC rates in terms of total citations and lower JSC rates in terms of citable items than those published in 2018. Citable items of most journals published in 2019 get fewer total citations than those published in 2018 (Figure 5(b)). The differences in the ratios of intradisciplinary citations and interdisciplinary citations to total citations are not obvious (Figure 5(c)).

**Figure 6:**
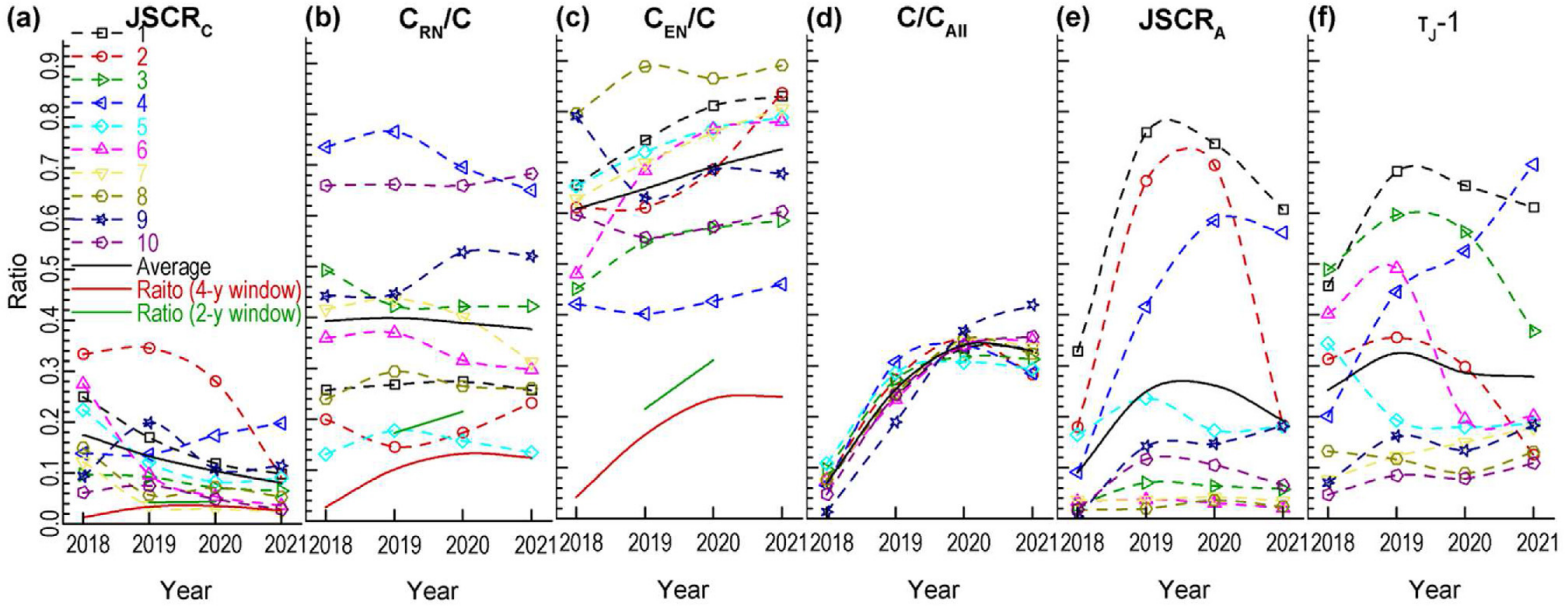
Dynamics of components and influence factors of journal self-citation and citation. **(a)** JSCR_C_. **(b)** C_RN_/C. **(c)** C_EN_/C. **(d)** C/C_All_. **(e)** JSCR_A_. **(e)**$\tau_{J}$-1 (JSCR_C_: journal self-citation rate (total citations). C_RN_: intradisciplinary non-self-citations. C: total citations in a year, four-year window or two-year window. C_EN_: interdisciplinary non-self-citations. C_All_: total citations in four-year window. JSCR_A_: journal self-citation rate (citable items). $\tau_{J}$: ratio of times cited to citing articles.).

The values of related indicators in different years (2018–2021) were compared for the citable items published by 10 selected journals in 2018 (Figure 6). The 10 journals were selected from the 10 groups of selected journals in Table 2 and the related data was obtained in T5. Among the six indicators, the ratio of citations in a year to total citations in the four-year window was the most consistent indicator (Figure 6(d)) and the JSC rate in terms of citable items was the most inconsistent indicator (Figure 6(e)) for different journals.

Regarding the average values of the indicators in a year (black real lines in Figure 6), the JSC rate in terms of total citations in a year got the highest value in the year when the citable items were published (Figure 6(a)). The ratios of intradisciplinary non-JSCs to total citations were almost at the same level (Figure 6(b)) and the ratio of interdisciplinary non-JSC to total citations would still increase in the third year after the citable items were published (Figure 6(c)). The citation reached its highest proportion in the second year but its proportion in the third year was still high (Figure 6(d)). The JSC rate in terms of citable items and the RTCA mostly matched the two-year window in which it got the two highest values (Figures 6(e) and (f)).

We also showed the proportions of JSCs, intradisciplinary non-JSCs and interdisciplinary non-JSCs in total citations in the four-year window (red real lines in Figures 6(a)–(c)). The dynamic of JSC was the quickest and the dynamic of interdisciplinary non-JSC was the slowest. In addition, interdisciplinary non-JSC occupies the advantage position in all years.

If only regarding the proportions of JSCs, intradisciplinary non-JSCs and interdisciplinary non-JSCs in total citations in the two-year window for JIF calculation (2019 and 2020, green real lines in Figures 6(a)–(c)), the interdisciplinary non-JSCs were also averagely the majority of citations, 31.1% in 2020 and 21.5% in 2019. The intradisciplinary non-JSCs were in the middle, 21.5% in 2020 and 17.2% in 2019. JSCs were the least, 4.4% in 2020 and 4.3% in 2019.

In Figure 6, we can find that most journals were more cited by interdisciplinary citations. The situations are different for the journal from IEEE (No.4 journal) and the journal from one-publishers with 4–5 WoSCs (No.10 journal) which got more intradisciplinary citations.

### 3.5 Relations between citation and number of articles

We analyzed the correlations between the related citations and the number of articles regarding the 100 selected journals in T5 (Table 5). Regarding the same citing articles, the changes in citable items, $n_{A,i-1}/n_{A,i-2}$, have strong significant correlations with the changes in the related citations, $T_{i,i-1}/T_{i,i-2}$. Regarding the same citable items, the changes in citing articles, $n_{A,i}/n_{A,i-1}$, have weak or even negative correlations with the changes in the related citation, $T_{i,i-2}/T_{i-1,i-2}$. In most cases, the simultaneous changes in both citable items and citing articles, $n_{A,i-1}/n_{A,i-2}$ and $n_{A,i}/n_{A,i-1}$, have strong significant correlations with the changes in its related citations, $T_{i,i-1}/T_{i-1,i-2}$.

**Table 5:** Correlations between citations and numbers of articles in selected journals regarding two-year window.

*rho*	$\frac{n_{A,2016}}{n_{A,2015}}$	$\frac{n_{A,2017}}{n_{A,2015}}$	$\frac{n_{A,2017}}{n_{A,2016}}$	$\frac{n_{A,2018}}{n_{A,2016}}$	$\frac{n_{A,2018}}{n_{A,2017}}$	$\frac{n_{A,2019}}{n_{A,2017}}$	$\frac{n_{A,2019}}{n_{A,2018}}$	$\frac{n_{A,2020}}{n_{A,2018}}$	$\frac{n_{A,2020}}{n_{A,2019}}$
$T_{2017,2016}/T_{2017,2015}$	**.65**∗∗	.57∗∗	.21∗	.21∗	.14	.19	.14	-.02	.08
$T_{2018,2017}/T_{2018,2016}$	.03	.39∗∗	**.47**∗∗	.37∗∗	.13	.11	.15	-.13	.00
$T_{2019,2018}/T_{2019,2017}$	.15	.17	.14	.43∗∗	**.55**∗∗	.33∗∗	.61∗∗	.09	.20∗
$T_{2020,2019}/T_{2020,2018}$	.26∗∗	.16	-.01	-.03	.09	.65∗∗	**.53**∗∗	.09	.49∗∗
$T_{2017,2015}/T_{2016,2015}$	.02	.04	**.04**	-.01	.05	-.00	.04	.17	.13
$T_{2018,2016}/T_{2017,2016}$	.04	-.03	-.03	.11	**.11**	-.06	.09	.16	.08
$T_{2019,2017}/T_{2018,2017}$	.11	.03	.00	-.04	-.06	-.11	**-.17**	.15	.00
$T_{2020,2018}/T_{2019,2018}$	-.08	-.04	.05	.02	-.09	-.14	-.15	.02	**-.06**
$T_{2017,2016}/T_{2016,2015}$	**.56**∗∗	**.51**∗∗	**.21**∗	.17	.14	.10	.09	.07	.11
$T_{2018,2017}/T_{2017,2016}$	.06	.42∗∗	**.48**∗∗	**.41**∗∗	**.15**	.16	.22∗	-.03	.10
$T_{2019,2018}/T_{2018,2017}$	.20	.19	.14	.46∗∗	**.59**∗∗	**.27**∗∗	**.57**∗∗	.14	.18
$T_{2020,2019}/T_{2019,2018}$	.20	.14	.04	-.01	.04	.55∗∗	**.42**∗∗	**.13**	**.48**∗∗

$n_{A,i}$: numbers of articles of journals in *i*th year.

$T_{i,j}$: times cited from articles in *i*th year citing articles in *j*th year.

Spearman, two-tailed.

∗ significant at level of 0.05.

∗∗ significant at level of 0.01.

The above correlations fit our EOM model in Eqs. (7), (8), (9), and (10). According to these equations, citing articles are positively associated with JSC and are negatively associated with intradisciplinary non-JSC. Citable items are positively associated with all components of citation.

### 3.6 Calculational results of average values of JIFs

**Figure 7:**
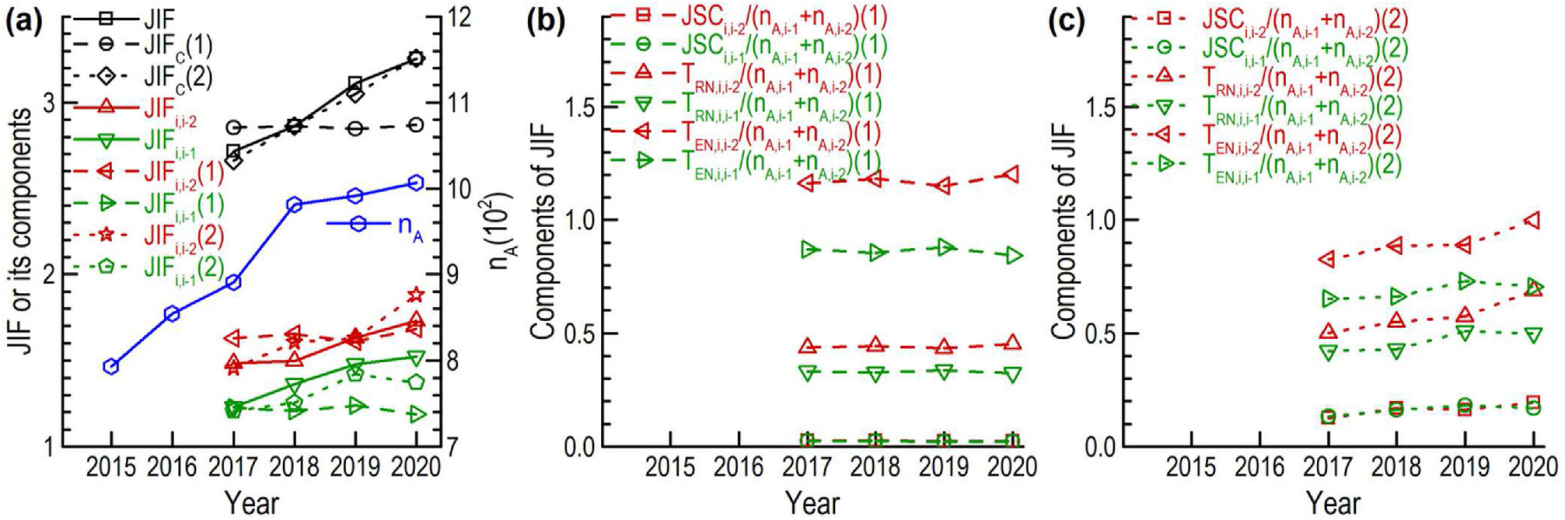
Comparison of statistical and calculational results of average values of JIFs and their components in 2017–2020 for 100 selected journals in T5. **(a)** Number of articles, JIF and its components. **(b)** Components of calculational JIF without increment effects of number of articles (1). **(c)** Components of calculational JIF with increment effects of number of articles (2) (T5: data time in October–December 2021. JIF: statistical results of journal impact factor. JIF_C_: calculational results of journal impact factor. JIF_i,i-2_ or JIF_i,i-1_: component of JIF contributed by citations in *i*th year citing articles in (*i*-2)th or (*i*-1)th year. n_A_: number of articles in a year. n_A,i-1_ or n_A,i-2_: number of citable items in (*i*-1)th or (*i*-2)th year. JSC_i,i-2_ or JSC_i,i-1_: component of JIF contributed by journal self-citations (times cited) in *i*th year citing citable items in (*i*-2)th or (*i*-1)th year. T_RN,i,i-2_ or T_RN,i,i-1_: component of JIF contributed by intradisciplinary non-self-citations (times cited) in *i*th year citing citable items in (*i*-2)th or (*i*-1)th year. T_EN,i,i-2_ or T_EN,i,i-1_: component of JIF contributed by interdisciplinary non-self-citations (times cited) in *i*th year citing citable items in (*i*-2)th or (*i*-1)th year).

The results mentioned in Section 3.5 motivated an attempt to calculate the average values of JIFs of these 100 selected journals in 2017–2020 (T5, Table 1) based on the EOM model. The calculation process is shown in Table 6 and the calculation results are shown in Figure 7.

**Table 6:** Citations and related values regarding the two-year window of journal impact factor.

	i−j=2	i−j=1	Values
$SP_{i}$	$a_{i}n_{0}$		$a_{2015-2020}$ = 2.64, 2.85, 2.97, 3.27, 3.30, 3.36 α = 1 or 2.9 (without or with increment effects) β = 1 or 0.8 α (without or with increment effects) γ = 1 or 0.6 α (without or with increment effects) d = 1.18 e = 1.41 f = 1.44 $\frac{f_{JS}}{f_{EN}}$ = 0.46 $\frac{f_{RN}}{f_{EN}}$ = 0.41 $f_{EN}b$ = 1.68 or 0.58 (without or with increment effects) $n_{0}$ = 300 $P_{2017-2020}$ = 14916, 15555, 17312, 18779 $\tau_{J}$ = 1.25
$SP_{j}$	$a_{i-2}n_{0}$	$a_{i-1}n_{0}$	
$p_{JS,i}$	${\left(a_{i}\right)}^{\gamma }n_{0}/P_{i}$		
$JSC_{i,j}$	$\frac{f_{JS}b{\left(a_{i-2}\right)}^{\alpha }n_{0}{\left(a_{i}\right)}^{\gamma }n_{0}}{P_{i}}d$	$\frac{f_{JS}b{\left(a_{i-1}\right)}^{\alpha }n_{0}{\left(a_{i}\right)}^{\gamma }n_{0}}{P_{i}}$	
$T_{RN,i,j}$	$f_{RN}b{\left(a_{i-2}\right)}^{\beta }n_{0}\left(1-\frac{{\left(a_{i}\right)}^{\gamma }n_{0}}{P_{i}}\right)e$	$f_{RN}b{\left(a_{i-1}\right)}^{\beta }n_{0}\left(1-\frac{{\left(a_{i}\right)}^{\gamma }n_{0}}{P_{i}}\right)$	
$T_{EN,i,j}$	$f_{EN}b{\left(a_{i-2}\right)}^{\gamma }n_{0}f$	$f_{EN}b{\left(a_{i-1}\right)}^{\gamma }n_{0}$	
$\underset{j}{\sum }SP_{j}$	$\left(a_{i-2}+a_{i-1}\right)n_{0}$		
$JSC_{i}$	$f_{JS}b\frac{{\left(a_{i}\right)}^{\gamma }n_{0}}{P_{i}}\frac{{\left(a_{i-1}\right)}^{\alpha }+{\left(a_{i-2}\right)}^{\alpha }d}{a_{i-1}+a_{i-2}}$		
$T_{RN,i}$	$f_{RN}b\left(1-\frac{{\left(a_{i}\right)}^{\gamma }n_{0}}{P_{i}}\right)\frac{{\left(a_{i-1}\right)}^{\beta }+{\left(a_{i-2}\right)}^{\beta }e}{a_{i-1}+a_{i-2}}$		
$T_{EN,i}$	$f_{EN}b\frac{{\left(a_{i-1}\right)}^{\gamma }+{\left(a_{i-2}\right)}^{\gamma }f}{a_{i-1}+a_{i-2}}$		

($a_{i}$: coefficients for articles of journals published in *i*th year (2015–2020). α,β,γ: adjustment coefficients for increment effects of numbers of articles on different components of citation. d,e,f: adjustment coefficients for different components of citation from citing articles in the year before last. $\;n_{0}$: number of articles of a reference journal. $T_{EN,i,j}$: average interdisciplinary non-self-citations (times cited) of journals in *i*th year citing citable items in *j*th year. $T_{RN,i,j}$: average intradisciplinary non-self-citations (times cited) of journals in *i*th year citing citable items in *j*th year. $JIF_{i}$: average journal impact factor of journals in *i*th year. $JSC_{i,j}$: average self-citations (times cited) of journals in *i*th year citing citable items in *j*th year. $P_{i}$: average number of articles in WoSCs. $p_{JS,i}$: average share of journals in *i*th year in WoSCs to which journals are allocated. $SP_{i}$: average number of articles in journals in *i*th year. $SP_{j}$: average citable items of journals in *j*th year.).

We supposed the journals were only allocated to one WoSC. The dynamics of different components of journal citation in Figures 6(a)–(c) were considered that the proportions of different components were different when the interval between the citing articles and the citable items are different (one year or two years). Three adjustment coefficients (*b*, *d* and *e*) were used when the citing articles cited the citable items published in the year before last. Here, it was supposed the values of $\tau_{J}$, $f_{RN}/f_{EN}$ and $f_{JS}/f_{EN}$ are constant for the citable items published in 2015–2019. We used the average values of $\tau_{J}$, $f_{RN}/f_{EN}$ and $f_{JS}/f_{EN}$ of the citable items published in 2018 in T5 (Table 4).

Because the actual effects of the number of articles on citation may be quite bigger than those being predicted in Eqs. (7), (8), (9), and (10), the other three adjustment coefficients (α, β and γ) were used for considering their effects on different components of citation. In this calculation, we firstly used the average value of statistical JIFs in 2018 to calculate the value of $f_{EN}b$. Then, we calculated the average values of JIFs in other years by supposing that the values of $f_{EN}b$ were constant in other years.

Without the increment effects of the number of articles, the calculational JIFs increased slightly from 2017 to 2020 which was far away from the statistical results. A small decrease even occurred in 2019 because the big increase in the number of articles in 2018 brought a big increase in the denominator for calculating the JIF in 2019. When considering the increment effects of the number of articles, the calculational JIFs constantly increase with the increase in the number of articles which agreed with the statistical results. In addition, the proportions of different components of journal citation also agreed with those of statistical results in Figures 6(a)–(c). The increment effects of the number of articles on JIF are stronger for JSC and citable items. Without the increment effects, the JSC rate in terms of total citations was only around 2% (Figure 7(b)). The JSC rate in terms of total citations rose to around 10% after considering the increment effects (Figure 7(c)).

The increment effects are introduced by complex dynamic interactions between different influence factors and different components of journal citation. The increment effects can be explained as follows:

• Preference-related self-citation and anomalous self-citation can directly increase JSCs per citable item. JSCs per citable item are also promoted by the increase in the journal's share in related disciplines regarding citing articles. The increased JSCs with a quick dynamic can promote non-self-citations with slow dynamics in the two-year window (Figures 6(a)–(c)).
• The increase in the number of articles, preference-related self-citation and anomalous self-citation can promote the multiple citations indicated by the RTCA which is of benefit to generate and benefit through a hot topic for promoting journal citation.
• The quick dynamic of JSC and the increased RTCA may avoid the appearance of the high JSC rate in the two-year window.

The increment effects of the number of articles on journal citation also include other aspects:

• With the constant increase in the available literature, authors increase the references in their articles also promoting citations per citable items.
• Publishing more articles may attract more academic concerns which may earn more submissions with high quality.

However, the differences between the statistical and calculational results still existed regarding the values of different components of JIFs (red and green lines in Figure 7(a)). The reasons can be explained as follows:

• Many journals are allocated to more than one WoSC, and therefore the increment effects of the number of articles may be different.
• The proportions of different components of citation are different regarding the citable items in different years. The usage of related coefficients in 2018 may introduce additional errors.
• The promotion of citation from JSC to non-JSCs was not explicitly discussed which may depend on the detail in the above two reasons.

In addition, the bonus of publishing more articles is limited when increasing the journal's shares in the related disciplines (WoSCs). According to Eqs. (8) and (10), and related equations in Table 6, the trend of the change in intradisciplinary non-JSC consists of two contrast aspects when increasing the journal's articles. The first is the positive aspect of citable items and the second is the negative aspect of the non-self proportion of citing articles. When the journal's share in the related disciplines exceeds a limit, the second negative aspect will become the main force resulting in the decrease of intradisciplinary non-JSCs per citable item and even the decrease of JIF. According to the related equations in Table 6, the intradisciplinary non-JSCs per citable item are roughly proportional to $\left(1-{\left(a_{i}\right)}^{\gamma }n_{A}/P_{i}\right){\left(a_{i}\right)}^{\beta -1}$. If using the values in Table 6, intradisciplinary non-JSCs per citable item will reach a peak when the number of articles is around 2,000.

This situation may be more serious for the journals that are more cited by intradisciplinary citations. In addition, for the journals with high JSC rates, increasing their articles may be dangerous. This action will firstly increase their JSCs which may result in big increases in their JSC rates.

### 3.7 Regarding some individual journals

**Figure 8:**
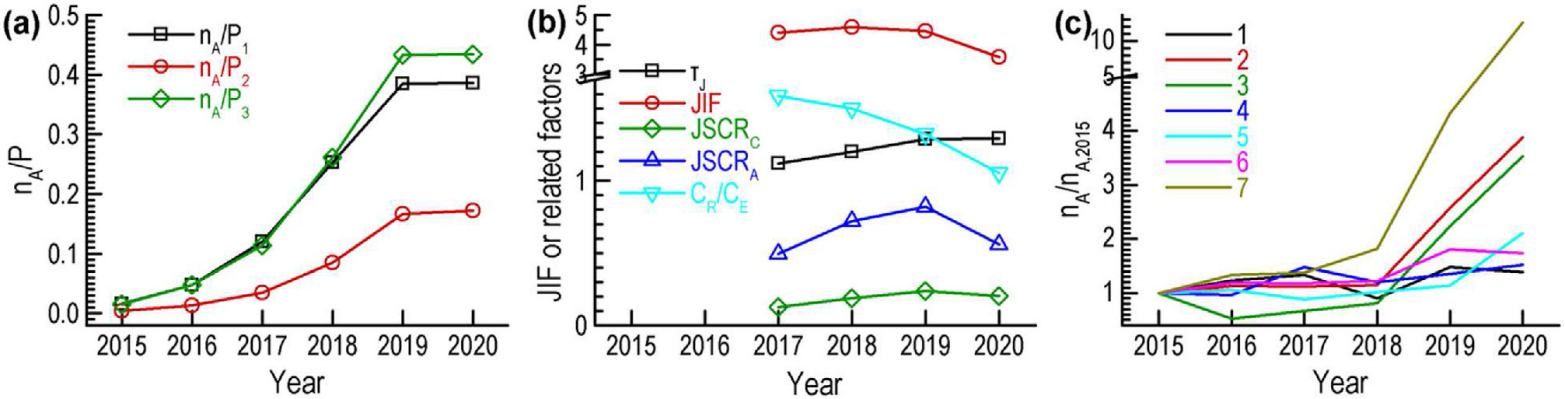
Numbers of articles, journal impact factors and other related factors of some individual journals. **(a)** n_A_/P of IEEE access. **(b)** JIF or related factors of IEEE Access. **(c)** n_A_/n_A,2015_ of suppressed journals in 2021 (n_A_: number of articles in 2015–2020. n_A, 2015_: number of articles in 2015. P_j_: number of articles of *j*th Web of Science category in 2015–2020. $\tau_{J}$: ratio of times cited to citing articles in two-year window. JIF: journal impact factor. JSCR_C_: journal self-citation rate (total citations) in two-year window. JSCR_A_: journal self-citation rate (citable items) in two-year window. C_R_: intradisciplinary citations in two-year window. C_E_: interdisciplinary citations in two-year window.).

Here, we gave an example, IEEE Access, which increased its articles from 249 to 17,935 in 2015–2020 (Figures 8(a) and (b)). IEEE Access is allocated to three WoSCs, Engineering Electrical Electronic, Computer Science Information Systems and Telecommunications. Its shares in these three WoSCs rose from all less than 2% to 17%, 39% and 43%, respectively. In contrast, its JIFs only increased in 2018 and then decreased in 2019 and 2020.

The main reason is the big decrease of intradisciplinary non-JSCs per citable item due to its high shares in the related disciplines and its stronger intradisciplinary citation. The ratio between intradisciplinary citations and interdisciplinary citations also decreased quickly (Figure 8(b)) due to its stable interdisciplinary citations per citable item. Its JSC rate in terms of total citations increased from 13% to 24% in 2017–2019 due to the increase in the number of its articles. Its JSC rate in terms of total citations was around 20% when the increase of its articles stopped in 2020. The RTCAs were also accorded with the changes in its articles in 2015–2020.

We also summarized the changes in the articles of the seven journals suppressed in 2021 due to coercive self-citation (Clarivate 2021). Among them, six journals increased their articles enormously in 2019 and/or 2020 (Figure 8(c)). The increases in their JSC rates due to the big increases in their articles may trigger the suppression criteria.

## 4 Discussion

### 4.1 Application of EOM to journal citation

The EOM was originally applied to discuss the citation behaviors of the top journal publishers' articles concerning the articles’ journals, disciples and publishers (Zhou, 2021). When applying the EOM model to the individual journal, the fitting errors increased (see Section 3.3).

The big fitting errors can be explained in three aspects. Firstly, the fitting errors were small in the calculations of $f_{RN}/f_{EN}$, but were big in the calculations of $\left(f_{JS}-f_{RN}\right)/f_{EN}$ (Table 4). It showed consistent behaviors of non-self-citation but inconsistent behaviors of self-citation for different journals which may be due to their different levels of anomalous self-citation and regular preference-related self-citation. Secondly, the citations of a journal are more sensitive to the citations of an individual article than those of a top journal publisher since the top journal publisher usually has a lot of journals and articles. And therefore, the fitting errors are often bigger when considering an individual journal. When applying the EOM model to the journals with more articles, the fitting error became smaller (Figure 3(a)). Thirdly, because some journals are allocated to more than one WoSC, the citation behaviors of journals may be varied according to the numbers of their WoSCs. When the journals were grouped in terms of the numbers of the journals’ WoSCs, the fitting errors also became smaller (Figure 3(b)).

When applying the EOM model to calculate JIFs in different years where the dynamics should be stressed for the number of articles and components of citation (see Section 3.6), the differences between the statistical and calculational results became quite big (Figures 7(a) and (b)). After considering the increment effects of the number of articles, the calculational results fitted not only the statistical JIFs but also the statistical results of different components of journal citation (Figures 7(b) and (c)). The difference between them can also be explained reasonably. We can also explain the situations of some individual journals which increased their articles recently (see Section 3.7).

In general, the EOM model is still applicable for calculating journal citation when the increment effects of the number of articles are considered. It should be indicated that the values of different parameters in Table 6 may be quite diversified for different journals. The values related to the increment effects will be determined regressively by comparing the statistics results and the calculational results of the concerned journal if we want to predict the JIF of an individual journal.

In the future, the EOM model can be improved by considering the discipline issues (the number of the journal's WoSCs) and the interaction of different components of citation in detail. In addition, some components of preference-related self-citation, e.g. journal self-citation introduced by authors' preference for journals, will be quantitatively discussed by considering the migration of author self-citation to JSC.

### 4.2 How journal citation is influenced

**Figure 9:**
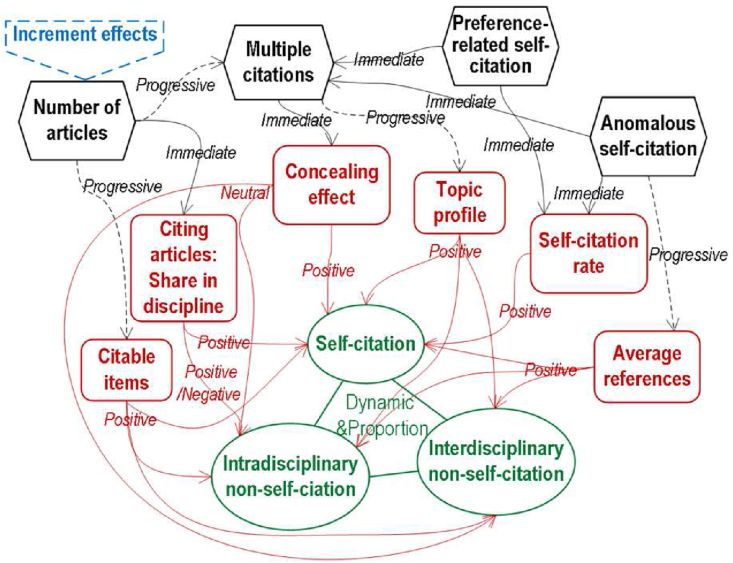
Relations between influence factors, journal self-citation and journal non-self-citations.

Here, our study revealed the dynamical interactions of influence factors (the number, discipline profile and topic profile of articles, multiple citations, preference-related self-citation and anomalous self-citation) and components of journal citation (JSC, intradisciplinary non-JSC and interdisciplinary non-JSC) (Figure 9).

Regarding the two-year window of JIF and its neighboring years, JSC has the quickest dynamic and interdisciplinary non-JSC has the slowest dynamic. In addition, most journals are more cited by interdisciplinary citations rather than intradisciplinary citations.

The change in the number of articles will first influence JSC and intradisciplinary non-JSC, by changing the journal's shares in related disciplines regarding citing articles. Then, the influence of changing the number of articles will be gradually presented to change all components of journal citation when the published articles sever as citable items with time. On the other hand, preference-related self-citation and anomalous self-citation will immediately promote JSC by increasing the self-citation rate. Besides, anomalous self-citation will progressively promote all components of journal citation by increasing average references.

The increase in articles can progressively promote multiple citations while preference-related self-citation and anomalous self-citation can increase the multiple citations immediately. The multiple citations, indicated by the RTCA, may progressively influence journal self-citation and citation in two following ways. Firstly, a high RTCA may result in a high skewness of the topic profile where it becomes easy for a journal to generate and benefit through hot topics. Secondly, the RTCA indicates the strength of concealing JSC since JSC is available on WoS regarding citing articles and JIF is calculated regarding times cited. With the same apparent JSC, the higher the RTCA of a journal is, the higher its actual JSC in terms of times cited is.

The dynamical interactions finally result in the increment effects of the number of articles on JIF when the journal's articles increase continuously. When the journal's shares in related disciplines are low, the increment effects often result in an increase in JIF with the increase in the number of articles.

However, intradisciplinary non-JSCs per citable item will decrease after the journal's share in related disciplines reaches a limit. When the number of articles becomes much higher, even JIF will decrease. The process may be quicker for the journal with a stronger intradisciplinary citation.

### 4.3 JSC in terms of total citations or citable items

Presently, the level of JSC is usually evaluated based on the ratio of JSCs to total citations in the related time window which continuously decreased recently (Figure 1(i)). A big increase in the JSC rate in terms of citable items occurred in T2 for the citable items published in 2017 (Figure 1(j)) mainly due to the obvious increase in the articles published in 2018 and the quick dynamic of JSC. It also resulted in a big increase in citations per citable item in T2 (Figure 1(g)) due to its strong correlation with journal citation (Table 3).

In addition, the journal with a quicker dynamic of JSC usually gets a higher level of self-citation in the same year when the citable items are published. Because this part of self-citation is before the two-year window, it will not be considered in the calculation of the JSC rate and JIF. However, it can promote the availability of the citable items which is of benefit to the citation of them in the following two-year window. The values of the JSC rate in terms of citable items were quite different for different journals as 0–3.40 (T1), 0–4.81 (T2) and 0–2.94 (T3), respectively.

We have to indicate the fact that coercive self-citation directly increases the JSC rate regarding citable items rather than the JSC rate regarding total citations. The JSC rate regarding total citations depends on the dynamic interactions of different influence factors and different components of citation in the related time window. Our full-scale statistical analysis showed strong significant correlations between the JSC rate in terms of citable items and journal citation and negative significant correlations between the JSC rate in terms of total citations and journal citation (Table 3). The strong significant correlation between the JSC rate in terms of citable items and journal citation indicates the effectiveness of JSC for promoting citation. The negative significant correlations can be explained by two aspects. With the same self-citation rate in terms of citable items, the lower the JIF of a journal is, the lower its JSC rate in terms of total citations is (Gorski et al., 2021). Secondly, the journal may have a high motivation to promote citation with coercive self-citation if its impact factor is at a low level. Present suppression policy concentrating on the JSC rate in terms of total citations may finally punish the failure to promote journal citation by using coercive self-citation. We suggest that the ratio of JSCs to citable items should also be considered to evaluate the level of self-citation of a journal. Although Clarivate started to annually warn about the excessive “advertisement” of some journals which published abnormal articles (usually review articles) with extra high JSCs, it is not enough.

## 5 Conclusion

Our statistical results showed constant increases in articles and citations versus a constant decrease in the self-citation rate in terms of total citations by considering citable items published in 2016–2018. Journal citation frequencies have strong correlations with JSC rates in terms of citable items, significant correlations with numbers of journals’ articles and negative significant correlations with JSC rates in terms of total citations. The equal opportunity model is applicable in the calculation of journal citation by considering the effects of different influence factors, namely, the number of articles, the discipline and topic profiles of articles, multiple citations, preference-related self-citation and anomalous self-citation. The differences between the statistical and calculational results can be reasonably explained.

Journal self-citation and citation are determined by the dynamic interactions of influence factors and components of citation. Firstly, the number of articles, the discipline and topic profiles of articles, multiple citations, preference-related self-citation and anomalous self-citation are interacted to influence the components of journal citation, namely, JSC, intradisciplinary non-JSC and interdisciplinary non-JSC. Secondly, the influences are strong dynamics within the two-year window and its neighboring years. Thirdly, the differences in the components of journal citation and the journal's shares in disciplines may vary the result of the interactions. In general, JSC has the quickest dynamic and interdisciplinary non-JSC has the slowest dynamic where interdisciplinary citations are higher than intradisciplinary citations for most journals regarding the two-year window and its neighboring years.

The complex dynamic interactions finally result in the increment effects of the number of articles on journal citation which is stronger for JSC and citable items. The increase in a journal's articles will increase JSCs and interdisciplinary citations per citable item due to the increment effects. When the journal's shares in its allocated disciplines are related low, it will also increase its intradisciplinary non-JSCs per citable item. Finally, the JIF increases continuously with the increase in its articles.

The increase can be reversed for intradisciplinary non-JSC when the journal's shares in related disciplines become high enough which may finally decrease its impact factor. This process becomes quicker for the journal with a stronger intradisciplinary citation.

Our calculational results of the average JIFs of the selected 100 journals agree with the related statistical results when the increment effects are considered. We can explain the changes in self-citation and citation of a journal with a strong intradisciplinary citation, IEEE Access, which enormously increased its articles recently. We also discussed the related situations of several journals suppressed in 2021 due to coercive self-citation regarding the changes in the numbers of their articles in 2015–2020.

It is also suggested that the self-citation rate in terms of citable items should be considered as an indicator for judging the level of JSC.

## Declarations

### Author contribution statement

Yangping Zhou, Ph. D: Conceived and designed the experiments; Performed the experiments; Analyzed and interpreted the data; Contributed reagents, materials, analysis tools or data; Wrote the paper.

### Funding statement

This work was supported by 10.13039/501100013076National Major Science and Technology Projects of China [ZX069].

### Data availability statement

The datasets generated during the current study are available at https://github.com/zhouyp97/JSC-JC-EOM.

### Declaration of interest’s statement

The authors declare no conflict of interest.

### Additional information

No additional information is available for this paper.
